# *Lilium regale* Wilson WRKY3 modulates an antimicrobial peptide gene, *LrDef1*, during response to *Fusarium oxysporum*

**DOI:** 10.1186/s12870-022-03649-y

**Published:** 2022-05-24

**Authors:** Zie Wang, Jie Deng, Tingting Liang, Linlin Su, Lilei Zheng, Hongjun Chen, Diqiu Liu

**Affiliations:** Faculty of Life Science and Technology, Kunming University of Science and Technology, Number 727 Jing Ming South Road, Chenggong District, Kunming, 650500 China

**Keywords:** *Lilium regale,*, WRKY transcription factor,, *Fusarium oxysporum*,, Transcriptional regulation,, Defensin

## Abstract

**Background:**

WRKY transcription factors (TFs) play vital roles in plant growth and development, secondary metabolite synthesis, and response to biotic and abiotic stresses. In a previous transcriptome sequencing analysis of *Lilium regale* Wilson, we identified multiple WRKY TFs that respond to exogenous methyl jasmonate treatment and lily Fusarium wilt (*Fusarium oxysporum*).

**Results:**

In the present study, the WRKY TF *LrWRKY3* was further analyzed to reveal its function in defense response to *F. oxysporum*. The LrWRKY3 protein was localized in the plant cell nucleus, and *LrWRKY3* transgenic tobacco lines showed higher resistance to *F. oxysporum* compared with wild-type (WT) tobacco. In addition, some genes related to jasmonic acid (JA) biosynthesis, salicylic acid (SA) signal transduction, and disease resistance had higher transcriptional levels in the *LrWRKY3* transgenic tobacco lines than in the WT. On the contrary, *L. regale* scales transiently expressing *LrWRKY3* RNA interference fragments showed higher sensitivity to *F. oxysporum* infection. Moreover, a *F. oxysporum*-induced defensin gene, *Def1*, was isolated from *L. regale*, and the recombinant protein LrDef1 isolated and purified from *Escherichia coli* possessed antifungal activity to several phytopathogens, including *F. oxysporum*. Furthermore, co-expression of *LrWRKY3* and the *LrDef1* promoter in tobacco enhanced the LrDef1 promoter-driven expression activity.

**Conclusions:**

These results clearly indicate that *LrWRKY3* is an important positive regulator in response to *F. oxysporum* infection, and one of its targets is the antimicrobial peptide gene *LrDef1*.

**Supplementary Information:**

The online version contains supplementary material available at 10.1186/s12870-022-03649-y.

## Background

WRKY transcription factors (TFs), the key regulators of plant response to biotic and abiotic stresses, constitute one of the largest TF families in plants. WRKY TFs possess a highly conserved domain comprising approximately 60 amino acid residues that contains one or two highly conserved short peptides (‘WRKYGQK’) at the N terminal, and a conserved zinc-finger motif of CX_7_CX_23_-HXC (C_2_HC) or CX_4-5_CX_22-23_HXH (C_2_H_2_) at the C terminal [1, 2]. The WRKY TF family can be classified into three groups based on the number of WRKY domains and the type of zinc-finger motif [1]. In general, group I contains two WRKY domains and a zinc-finger motif. Group II contains a WRKY domain and a C_2_H_2_ zinc-finger motif, whereas group III includes one WRKY motif and the zinc-finger domain C_2_HC. Group I WRKY TFs can be divided into two subgroups: the Ia subgroup possessing a C_2_H_2_ zinc-finger motif, and the Ib subgroup containing a C_2_HC zinc-finger motif. Group II WRKY TFs can be further subdivided into five subgroups, IIa, IIb, IIc, IId, and IIe, according to their phylogenetic relationships [3, 4].

WRKY TFs have been shown to be important regulators in plant defense response against a variety of pathogens. For example, 16 *CmWRKY* genes exhibit distinct expression patterns in melon (*Cucumis melo*) during *Podosphaera xanthii* infection [5]. In peony (*Paeonia lactiflora*) infected by *Alternaria tenuissima*, *PlWRKY65* expression is significantly enhanced, whereas reduced expression of *PlWRKY65* via virus-induced gene silencing enhances sensitivity to *Alternaria tenuissima* infection [6]. *Arabidopsis thaliana* plants expressing grape (*Vitis labrusca* × *V. vinifera*) *WRKY3* exhibit improved salt and drought tolerance at germination, seedling, and maturity stages. Compared with wild-type (WT) plants, *VlWRKY3* transgenic Arabidopsis lines also have increased resistance to *Golovinomyces cichoracearum* but increased sensitivity to *Botrytis cinerea* [7].

WRKY TFs self-regulate and cross-regulate with other TFs to control the defense response of plants to biotic stress. In rice (*Oryza sativa*), *OsWRKY45–2* enhances resistance to *Magnaporthe oryzae* by transcriptionally activating *OsWRKY13* [8]. The overexpression of oilseed rape (*Brassica napus*) *WRKY15* inhibits the transcriptional activation of *BnWRKY33*, which leads to increased plant susceptibility to *Sclerotinia sclerotiorum* [9]. *CaWRKY40b* in pepper (*Capsicum annuum*) exhibits positive feedback regulation at the transcriptional level by directly targeting the W box element in its own promoter; this gene plays a negative regulatory role in the defense response of pepper against *Ralstonia solanacearum*, thus reducing plant immunity [10].

Lily (*Lilium*) is one of most popular fresh-cut flowers but is vulnerable to Fusarium wilt disease caused by pathogenic *Fusarium* species, such as *F. oxysporum*. Fusarium wilt affects the yield and quality of lily bulbs and cut flowers and also causes withering and death of lily plants. In our previous study, we carried out a preliminary investigation of the molecular interaction mechanism between a wild lily (*L. regale* Wilson) and *F. oxysporum* and confirmed that *L. regale* has strong resistance to *F. oxysporum* [11]. Drawing on the results of our previous *L. regale* transcriptome sequencing analysis, 35 WRKY genes responsive to *F. oxysporum* were cloned from *L. regale* [12]. qRT-PCR analysis revealed that the expression levels of *LrWRKY*s were induced by the exogenous application of four signaling molecules, such as salicylic acid (SA), methyl jasmonate (MeJA), and ethylene (ETH). Furthermore, the functional analysis confirmed that LrWRKY2 is a positive regulator that mediates *L. regale* defense responses to *F. oxysporum* infection by regulating *chitinase 2* expression [13]. Compared with *LrWRKY2*, *LrWRKY3* (GenBank no. MW125548) showed higher expression levels in root, stem, leaf, and flower tissues and upon treatment with SA, MeJA, and ETH. Moreover, *LrWRKY3* exhibited more transcript accumulation in the *L. regale* roots during *F. oxysporum* infection than *LrWRKY2* did. WRKY TF family members regulate a complex defenses network in plants in response to biotic stress [14], and similar observations were made in *L. regale*.

In this study, we investigated the regulatory mechanism of *LrWRKY3* in defense response of *L. regale* to *F. oxysporum*. The onion (*Allium cepa*) epidermis cells without the chloroplast disturbance are large and easy to observe under microscope, thus they are commonly used for subcellular localization analysis of plant proteins. The transient expression in onion epidermal cells mediated by the *Agrobacterium tumefaciens* was used to analyze the subcellular localization of LrWRKY3. In addition, due to the current lack of a rapid and efficient method for the lily genetic transformation [15], *LrWRKY3* was heterologously expressed in tobacco (*Nicotiana tabacum*) for functional verification. It is widely known that tobacco is an important model plant for rapid confirmation of gene functions, and *F. oxysporum* can rapidly cause Fusarium wilt in tobacco [16]. In addition, the *LrWRKY3* function in defense response was explored in depth by transiently expressing its RNA interference (RNAi) fragment in *L. regale* scales. Finally, we isolated a *F. oxysporum*-responsive defensin gene (*LrDef1*, GenBank no. MZ872924) from *L. regale* and confirmed the resistance of *LrDef1* to *F. oxysporum*. Moreover, this study further verified that LrWRKY3 regulates *LrDef1* expression considering that the *LrDef1* promoter had a W-box and rarely known interaction between the WRKY TFs and defensin during response to phytopathogen infection in plants.

## Results

### The *L. regale* subgroup-IIa *WRKY3* gene was found to encode a nuclear protein

**Fig. 1:**
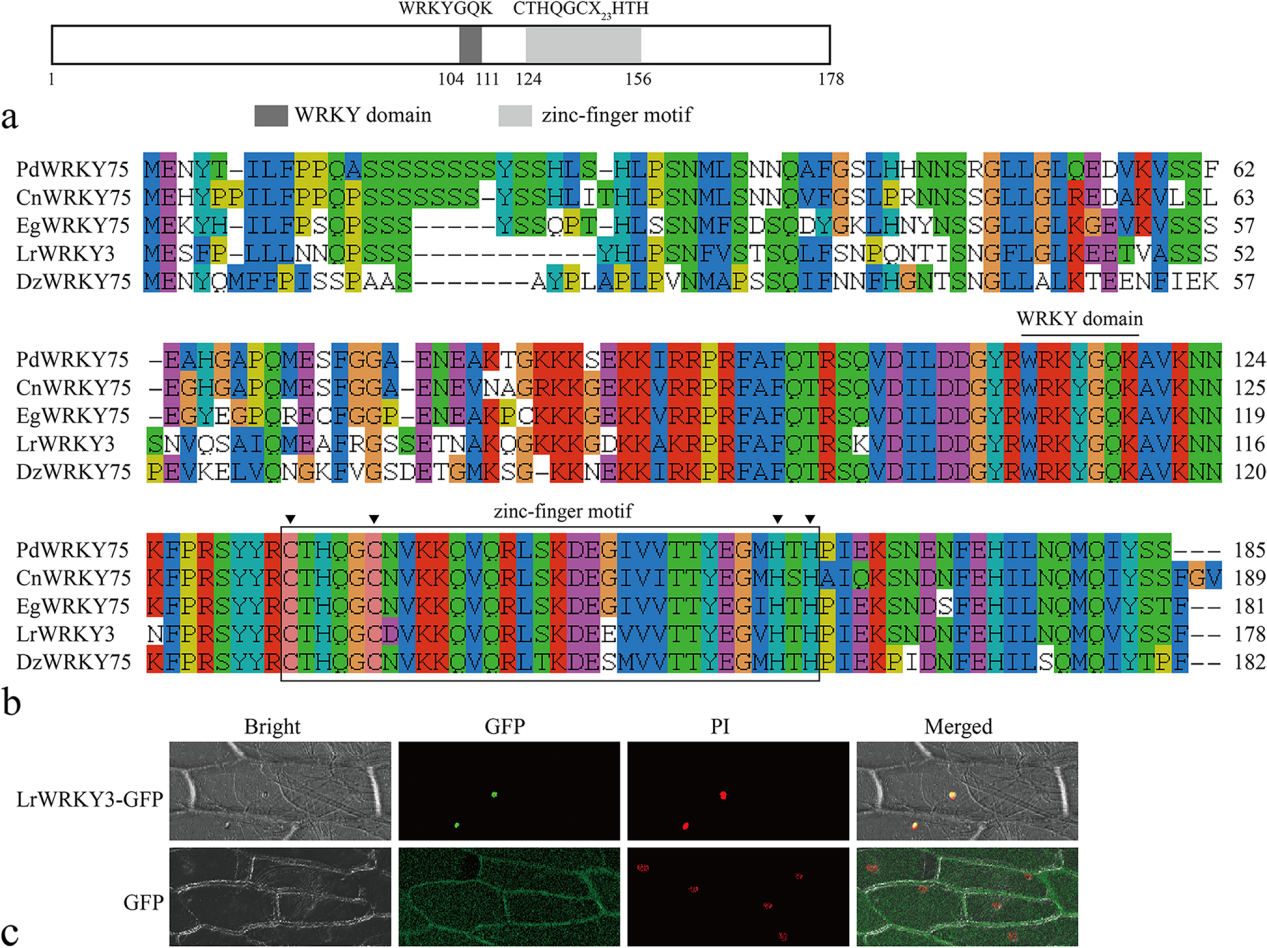
The protein analysis of LrWRKY3. **a** The protein structure analysis of LrWRKY3. **b** The multiple alignment of deduced LrWRKY3 amino acid sequences and four homologous sequences were performed with the ClustalW. **c** The transient expression LrWRKY3-GFP fusion protein in onion epidermal cells. It revealed that LrWRKY3 was localized in plant cell nucleus. GFP, fluorescent light; Bright, white light; PI, the nucleus was displayed by propidium iodide staining; Merged, overlaid of fluorescent light, white light and PI

In our previous study, we identified a series of WRKY genes from a Fusarium wilt-resistant wild lily (*L. regale*) by transcriptome sequencing. According to the gene expression pattern data, *LrWRKY3* was induced by *F. oxysporum* and responded to signaling molecule treatments [12]. In the present study, the function of *LrWRKY3* was therefore further characterized to understand the transcriptional regulation of WRKY in *L. regale* in response to Fusarium wilt. The full-length cDNA of *LrWRKY3*, which was found to be 722 bp in length with a 537-bp open reading frame (ORF), was predicted to encode a subgroup-IIa WRKY protein with 178 amino acid residues. The calculated molecular mass of the deduced LrWRKY3 was 20.3 kDa. According to our sequence analysis, LrWRKY3 contained a highly conserved ‘WRKYGQK’ sequence and a zinc-finger motif CX_4-5_CX_22-23_HXH (C_2_H_2_), which suggests membership in the IIa WRKY subgroup (Fig. 1a). The deduced amino acid sequence of *LrWRKY3* was highly similar to that of some WRKY proteins, including *Phoenix dactylifera* WRKY75 (GenBank no. XP_008809548.2), *Cocos nucifera* WRKY75 (KAG1370131.1), *Elaeis guineensis* WRKY75 (XP_010913024.1), and *Durio zibethinus* WRKY75 (XP_022740913.1) (Fig. 1b).

The result of PSORT prediction suggested that the protein encoded by *LrWRKY3* is localized in the plant cell nucleus. To confirm the subcellular location of LrWRKY3, a fusion expression vector of *LrWRKY3* and *green fluorescent protein* (*GFP*) was constructed and expressed in onion epidermal cells through *Agrobacterium tumefaciens*-mediated transformation. The green fluorescence signal of LrWRKY3-GFP fusion protein was exclusively distributed in the nuclei of onion epidermal cells and colocalized with the nuclear localization marker PI (Fig. 1c). In contrast, green fluorescence was detected throughout the entire onion epidermal cell in control. This result indicates that LrWRKY3 is localized in plant cell nucleus.

### Overexpression of *LrWRKY3* in tobacco increased resistance to *F. oxysporum* and enhanced transcription levels of some JA/SA signaling pathway genes, *PRs*, and *SODs*

**Fig. 2:**
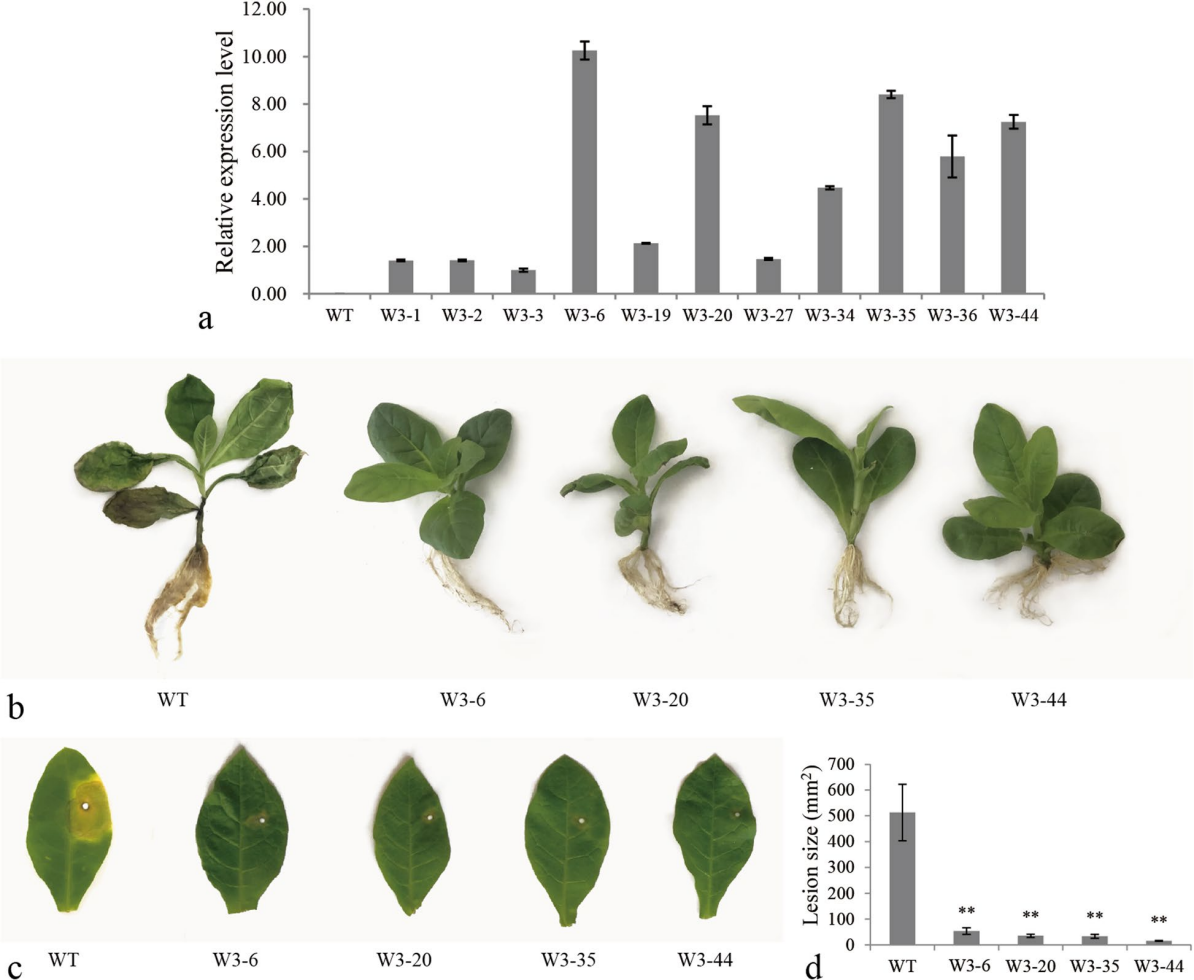
Gene expression and resistance analyses of *LrWRKY3* T_2_ generation transgenic tobacco lines. **a** The expression level of *LrWRKY3* in T_2_ generation transgenic tobacco by qRT-PCR. The *LrWRKY3* was stably expressed in transgenic tobacco lines. WT: wild-type tobacco; W3–1/2/3/6/19/20/27/34/35/36/44: *LrWRKY3* T_2_ transgenic tobacco lines. **b** The root inoculation assay revealed the enhanced resistance of four *LrWRKY3* T_2_ transgenic tobacco lines (W3–6/20/35/44) against *F. oxysporum*. **c** The leaf inoculation assay revealed the enhanced resistance of four *LrWRKY3* T_2_ transgenic tobacco lines (W3–6/20/35/44) against *F. oxysporum*. **d** The results of leaf inoculation showed that the lesion areas in transgenic tobacco were significantly smaller than that in the WT. The results were shown as average values calculated from three replicates and calculated by the 2^-ΔΔCt^ method and analyzed by the Student’s *t* test (** *p* < 0.01)

To further understand the biological function of *LrWRKY3*, the plant overexpression vector pCAMBIA2300s-*LrWRKY3* was constructed and transformed into WT tobacco leaf disks. A total of 50 T_0_ transgenic tobacco plants were obtained according to PCR analysis with *LrWRKW3-*specific primers. qRT-PCR was used to analyze the expression levels of *LrWRKY3* in leaves of 11 T_2_ generation transgenic lines (W3–1/2/3/6/19/20/27/34/35/36/44), with WT serving as a negative control. According to the results, *LrWRKY3* transcripts accumulated and were differentially expressed in the 11 transgenic tobacco lines (Fig. 2a). *LrWRKY3* transcription was highest in line W3–6, with a relative expression value of 10.25. *LrWRKY3* relative expression levels in W3–20, W3–35 and W3–44 were also high, and the expression value was 7.53, 8.40, and 7.25, respectively. These data indicate that *LrWRKY3* was stably expressed in T_2_ generation transgenic tobacco.

Four transgenic lines (W3–6/20/35/44) with high *LrWRKY3* expression and WT plants were inoculated with a spore suspension of *F. oxysporum* to evaluate their resistance to the pathogen. The roots of WT tobacco turned black and rotted, and whole leaves had wilted 7 days after inoculation (Fig. 2b). In contrast, the roots of T_2_ generation transgenic tobacco lines showed no evident disease symptoms, and the leaves were healthy, bright green, and non-wilted. The results of leaf inoculation experiments were consistent with these observations. The leaves of transgenic lines exhibited only slight deterioration and local yellowing near the inoculation wound, whereas leaves of WT were obviously more yellowed and decayed, with a larger damaged area, 7 days after inoculation (Fig. 2c–d). These results indicate that the overexpression of *LrWRKY3* in tobacco improves disease resistance to *F. oxysporum*.

**Fig. 3:**
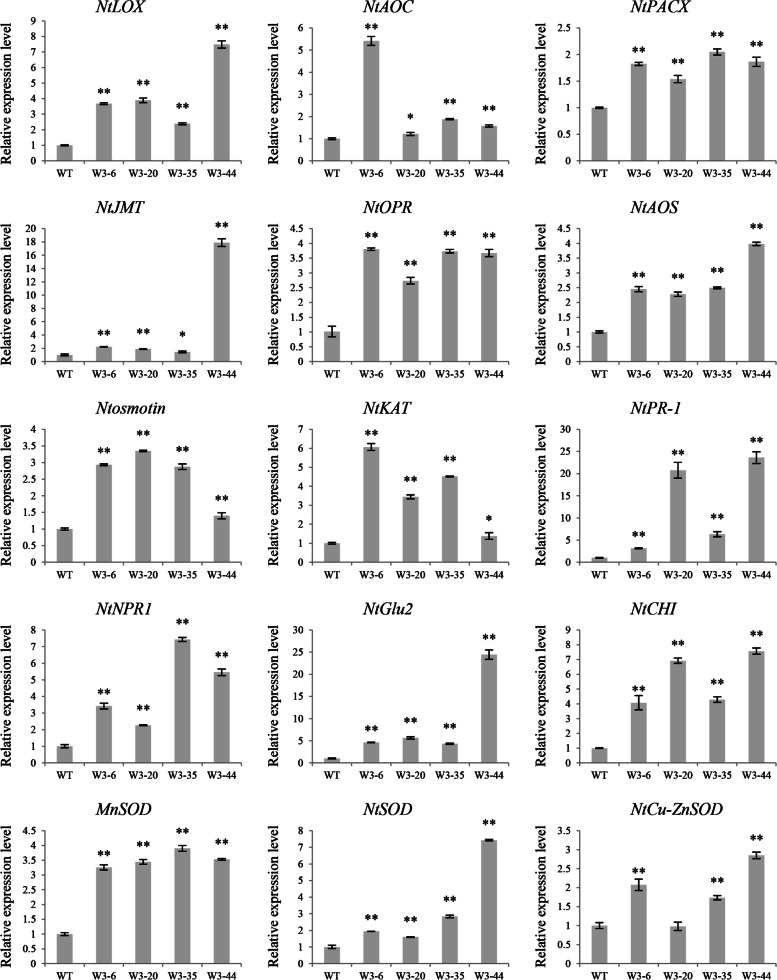
The expression levels of JA/SA signaling pathway-related genes, *PRs* and *SODs* were up-regulated in the *LrWRKY3* T_2_ transgenic tobacco lines. WT: wild-type tobacco; W3–6/20/35/44: *LrWRKY3* T_2_ transgenic tobacco lines. The results were shown as the average values calculated from three replicates and calculated by the 2^-ΔΔCt^ method and analyzed by the Student’s *t* test (** *p* < 0.01, * *p* < 0.05)

Transcription levels of some JA/SA signaling and defense-related genes in transgenic tobacco lines and WT tobacco were analyzed by qRT-PCR. As shown in Fig. 3, transcription levels of JA biosynthetic pathway-related genes, including *NtLOX*, *NtAOC*, *NtOPR*, *NtAOS*, *NtJMT*, *NtKAT*, and *NtPACX*, were obviously elevated in *LrWRKY3* transgenic lines compared with WT. The expression level of *NtJMT* was highest in line W3–44: 16.89-fold higher than in WT tobacco. Compared with their expressions in WT, *NtLOX* and *NtAOS* were up-regulated in W3–44 by 6.49- and 2.98-fold, respectively. Relative expression levels of the SA signaling pathway-related genes *NtPR1* and *NtNPR1* were increased in transgenic tobacco lines. The expression of *NtPR1* in line W3–44 was 24-fold that of WT, and the expression of *NtNPR1* in line W3–35 was 7-fold that of WT. In W3–44, the expression levels of pathogenesis-related protein (*PR*) genes *NtGlu2* and *NtCHI* were respectively 24- and 8.5-fold higher than that of WT. In addition, the expression level of *PR* gene *Ntosmotin* in line W3–20 was 3.4-fold higher than that in WT. Furthermore, antioxidant stress-related superoxide dismutase (*SOD*) genes, including *NtSOD*, *NtCu-ZnSOD*, and *MnSOD*, were all up-regulated in transgenic tobacco compared with WT. In conclusion, the overexpression of *LrWRKY3* in tobacco up-regulated the expressions of JA/SA signaling pathway-related genes, *PRs* and *SODs*.

### Transient expression of the *LrWRKY3* RNAi vector in *L. regale* scales increased susceptibility to *F. oxysporum*

**Fig. 4:**
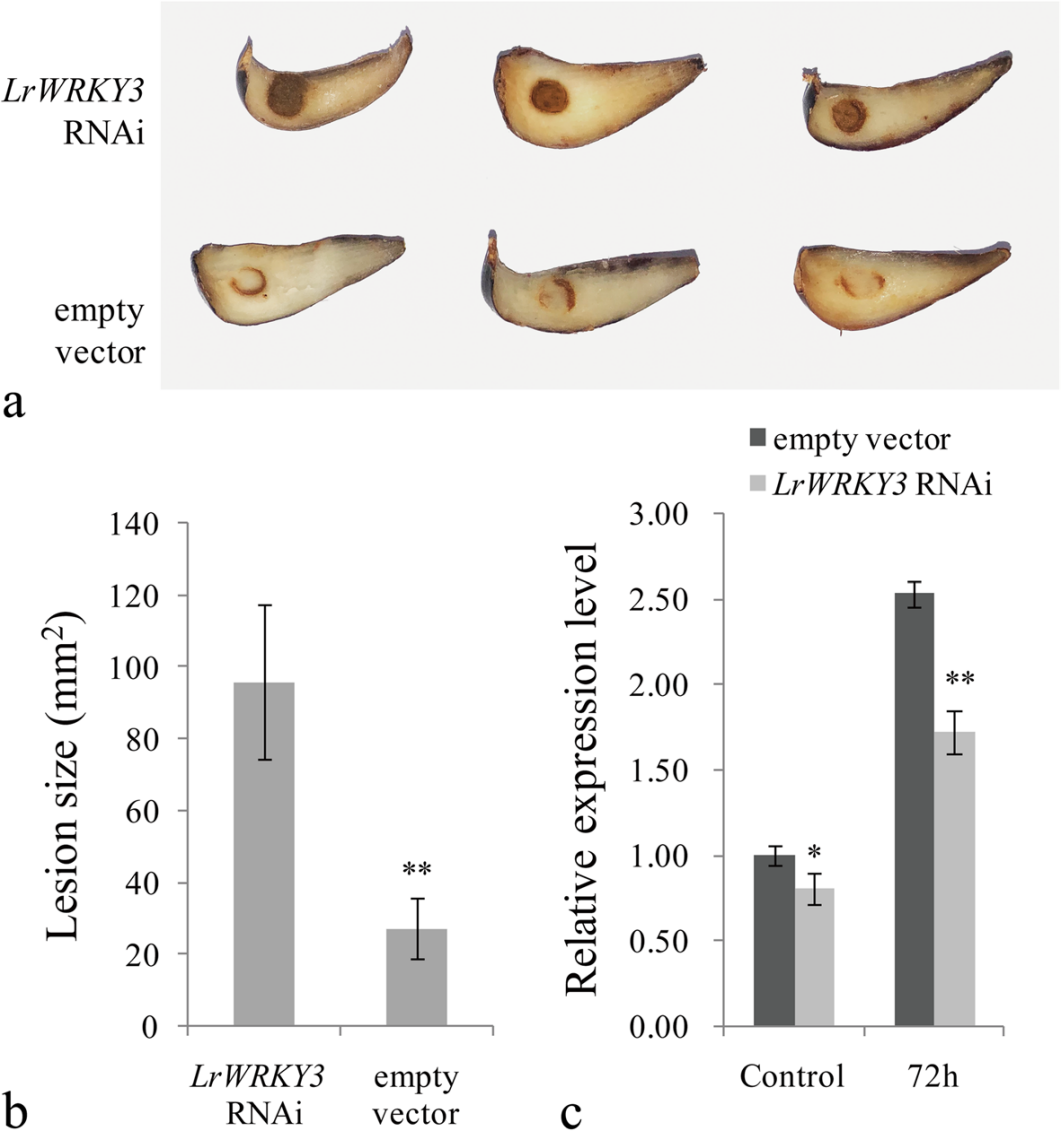
Analysis of *L. regale* scales after transient expressing the *LrWRKY3* RNAi vector. **a** The symptoms of *L. regale* scale after *F. oxysporum* inoculation, in which the *LrWRKY3* RNAi vector and the empty RNAi vector was expressed, respectively. **b** Measurements of scales lesions caused by *F. oxysporum* infection. The results were shown as the average values calculated from three replicates and the Student’s *t* test was used to analyze the statistical difference (** *p* < 0.01). **c** The expression level of *LrWRKY3* in *L. regale* scale transiently expressing the *LrWRKY3* RNAi construct was evaluated by qRT-PCR. The empty RNAi vector infected samples with *F. oxysporum* inoculation was the control of statistical analysis. The results were calculated by the 2^-ΔΔCt^ method and analyzed by the Student’s *t* test (** *p* < 0.01, * *p* < 0.05)

To further analyze the function of *LrWRKY3*, a *LrWRKY3* RNAi construct was transiently expressed in *L. regale* scales via *Agrobacterium tumefaciens* mediation, and the scales were then inoculated with *F. oxysporum* spore suspension. Obvious blackening and decay appeared on the infected scales expressing the *LrWRKY3* RNAi construct, but disease symptoms were much less evident on *L. regale* scales transformed with *Agrobacterium tumefaciens* containing an empty RNAi vector (Fig. 4a). Calculation of the damage area on *F. oxysporum*-infected *L. regale* scales confirmed that *L. regale* expressing the *LrWRKY3* RNAi fragment was less resistant to *F. oxysporum* infection than the control (RNAi empty vector) (Fig. 4b). In addition, qRT-PCR analysis revealed that the expression level of *LrWRKY3* in RNAi fragment-expressing *L. regale* was evidently lower than that in control scales, whether inoculated with *F. oxysporum* or not (Fig. 4c). These data clearly indicate that the decreased expression of *LrWRKY3* in *L. regale* scales enhanced sensitivity to *F. oxysporum*.

### The *L. regale* defensin gene *Def1* was revealed to be a *F. oxysporum* resistance gene

To explore the regulation of *PR* gene expression by LrWRKY3, a *L. regale* defensin gene, *LrDef1*, was cloned on the basis of transcriptome sequencing data (unpublished). The full-length cDNA of *LrDef1* was 501 bp, with a 225-bp coding region, and was predicted to encode a protein containing 74 amino acid residues. The predicted molecular weight of the deduced protein LrDef1 was approximately 8.06 kDa. The *LrDef1* promoter sequence (850 bp) was obtained by genome walking. The cDNA sequence of *LrDef1* and the sequence of its promoter fragment were given in the supplementary information (Additional file 1). Using the PlantCARE prediction program, we identified many *cis*-acting elements in the *LrDef1* promoter, including the TGACG motif (MeJA response element), W box (ET, SA, and MeJA response element), and ABRE (ABA response, hypersaline, and dark induction element), and a series of *cis*-elements involved in response to plant hormone signals and abiotic and biotic stresses. The prediction results of *cis*-acting elements in the promoter sequence of *LrDef1* were given in supplementary information (Additional file 2, Table S1).

**Fig. 5:**
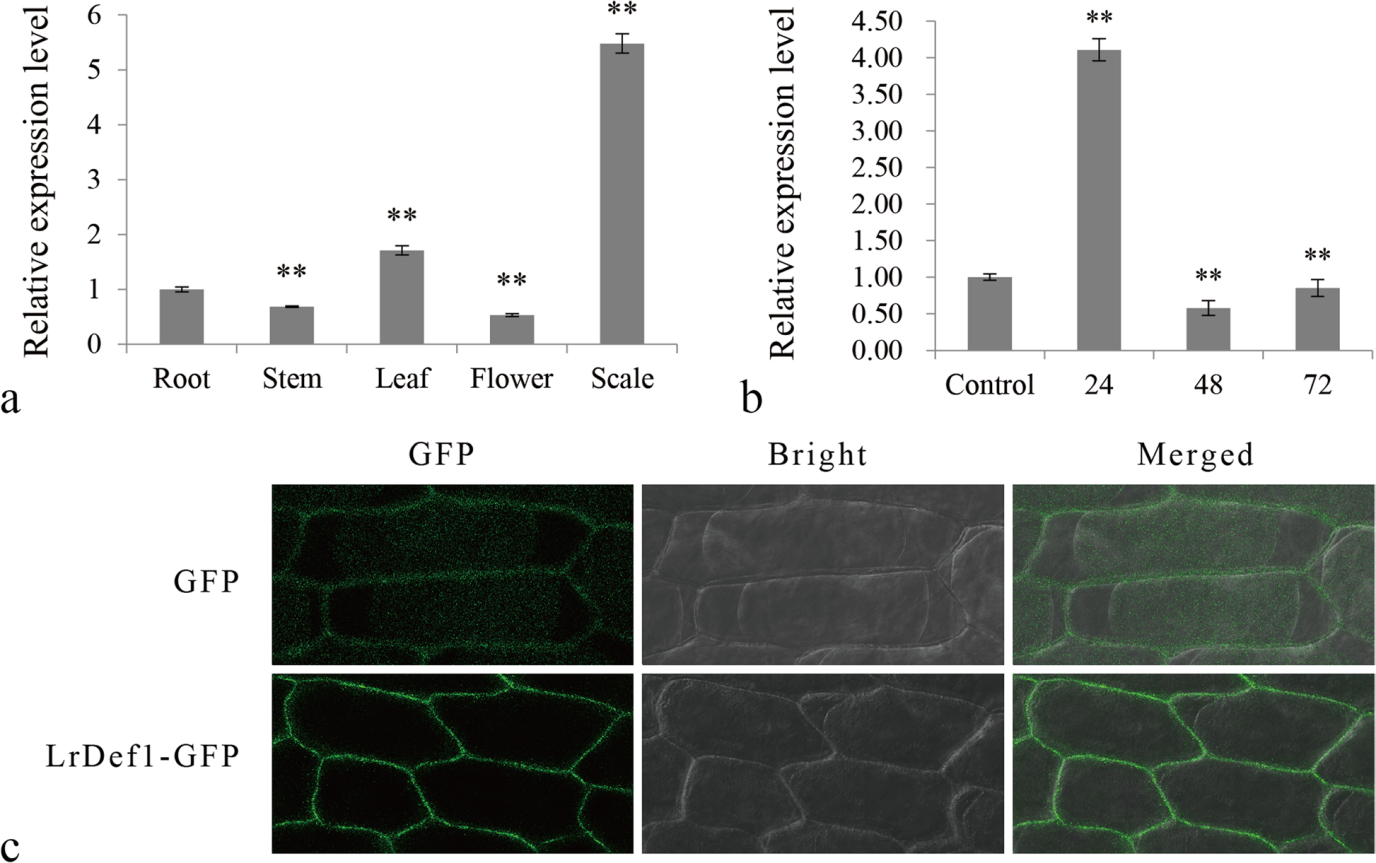
Expression profile analysis and subcellular localization analysis of LrDef1. **a** The expression levels of *LrDef1* in various tissues of *L. regale*. **b** The expression level of *LrDef1* in *L. regale* roots at different time points after *F. oxysporum* inoculation. **c** The transient expression of LrDef1-GFP fusion protein in onion epidermal cells revealed the LrDef1 localized in plant cell wall. GFP, fluorescent light; Bright, white light; Merged, overlaid of fluorescent and white light

In the qRT-PCR analysis, *LrDef1* transcripts were detected in *L. regale* roots, stems, leaves, flowers, and scales (Fig. 5a). In particular, *LrDef1* was strongly expressed in scales. After *F. oxysporum* inoculation, the expression of *LrDef1* in *L. regale* roots was rapidly induced, with a peak at 24 h (Fig. 5b) when the expression level was approximately 4.1-fold of the control. According to these results, *LrDef1* is a *F. oxysporum* infection-induced gene and is dominantly expressed in *L. regale* scales. An N-terminal signal peptide was detected in the deduced protein LrDef1, which was predicted to localize in plant cell wall, thus indicating that LrDef1 may be a secretory protein. Moreover, the subcellular localization of LrDef1 also examined by fusion expression with *GFP* in onion epidermal cells. The green fluorescence signal of fusion gene of *LrDef1* and *GFP* was distributed in the cell wall of onion epidermal cells (Fig. 5c). This result demonstrates that LrDef1 is located in plant cell wall as an extracellular protein.

**Fig. 6:**
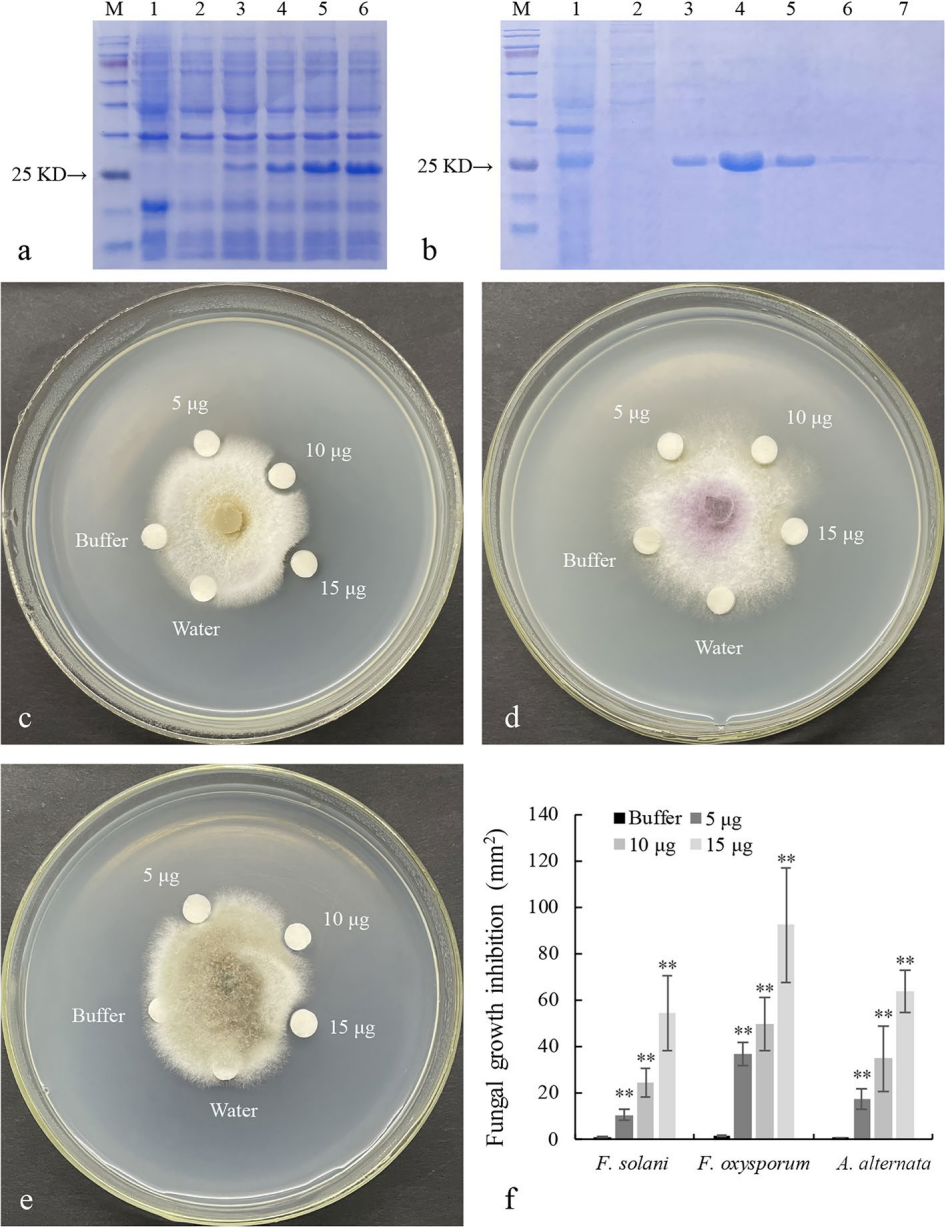
Expression, purification and antifungal assay of LrDef1 recombinant protein. **a** The induced expression of LrDef1 recombinant protein under 1 mM IPTG condition. M, protein marker; 1, the His tagged protein of empty vector pET-32(a) induced was detected after induction; 2, the expression of LrDef1 protein was detected without induction; 3–6, the expression of LrDef1 protein was detected at 2, 4, 6, and 8 h after induction, respectively. **b** The purification of LrDef1 recombinant protein. M, protein marker; 1, the supernatant after the *E. coli* was broken; 2, the precipitate after the *E. coli* was broken; 3–7, the purified LrDef1 recombinant protein with 50-, 100-, 150-, 200- and 250-mM imidazole washing buffer, respectively. **c**–**e** The LrDef1 recombinant protein has evident antifungal activity to *F. solani* (**c**), *F.oxysporum* (**d**) and *Alternaria alternata* (**e**)*.* **f** The fungal growth inhibition analysis showed the antifungal activities of protein to *F.oxysporum*, *F. solani* and *Alternaria alternata*. The results were shown as average values calculated from three replicates and calculated by the 2^-ΔΔCt^ method and analyzed by the Student’s *t* test (** *p* < 0.01)

The recombinant plasmid pET-32(a)-*LrDef1*-His containing a His-tag was transformed into *Escherichia coli* strain BL21 (DE3) for heterologous expression. As revealed by SDS-PAGE, the molecular mass of the induced His-LrDef1 fusion protein was consistent with its predicted size, 25 kDa (Fig. 6a). The recombinant protein was purified by Ni-NTA-sepharose (Sangon Biotech, China) column affinity chromatography and imidazole elution buffer (Fig. 6b) and then used in antifungal experiments. Original image of Fig. 6a and Fig. 6b were given in supplementary information (Additional file 3). As shown in Fig. 6c–e, the LrDef1 recombinant protein had different inhibitory effects on the mycelial growth of three fungi: *F. oxysporum*, *F. solani*, and *Alternaria alternata.* The recombinant protein had the strongest inhibitory effect on *F. oxysporum*, followed by *Alternaria alternata* and *F. solani*. Moreover, the antifungal activity increased along with the mass of LrDef1 recombinant protein (Fig. 6f).

**Fig. 7:**
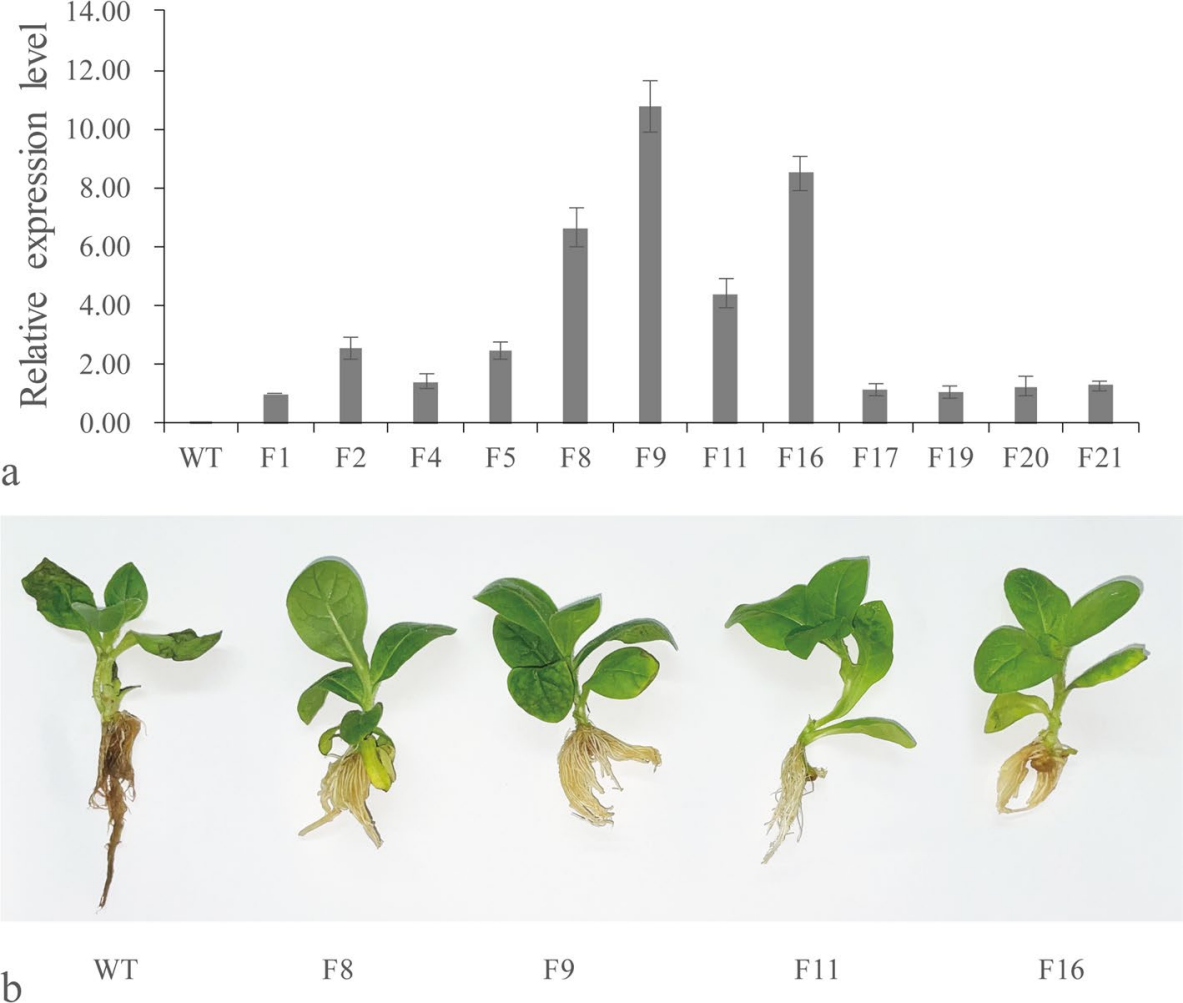
Gene expression and resistance analyses of *LrDef1* T_2_ generation transgenic tobacco lines. **a** The expression level of *LrDef1* in the T_2_ generation transgenic tobacco by qRT-PCR. The *LrDef1* was stably expressed in transgenic tobacco. WT: wild-type tobacco; F1/2/4/5/8/9/11/16/17/19/20/21: *LrDef1* T_2_ transgenic tobacco lines. **b** The roots inoculation assay revealed the enhanced resistance of four *LrDef1* T_2_ transgenic tobacco lines (F8/9/11/16) against *F. oxysporum*

PCR analysis with *LrDef1*-specific primers identified 35 T_0_ *LrDef1* transgenic tobacco plants. In addition, qRT-PCR was used to analyze the expression levels of *LrDef1* in 12 T_2_ generation transgenic lines (F1/2/4/5/8/9/11/16/17/19/20/21), and the result indicated that *LrDef1* was expressed in all transgenic lines (Fig. 7a). We selected four of the T_2_ generation *LrDef1* transgenic tobacco lines (F8/9/11/16) to study their resistance to *F. oxysporum*. Seven days after inoculation with *F. oxysporum* spore suspension (2 × 10^6^ spores/mL), WT tobacco roots were black and rotten, and some leaves had started to shrink. In contrast, the roots of transgenic tobacco lines were only slightly darkened, and the leaves were still fully extended (Fig. 7b). Overexpression of *LrDef1* in tobacco thus enhanced resistance to *F. oxysporum* infection.

### The LrWRKY3 recombinant protein specifically bound to the *LrDef1* promoter fragment with a W box

**Fig. 8:**
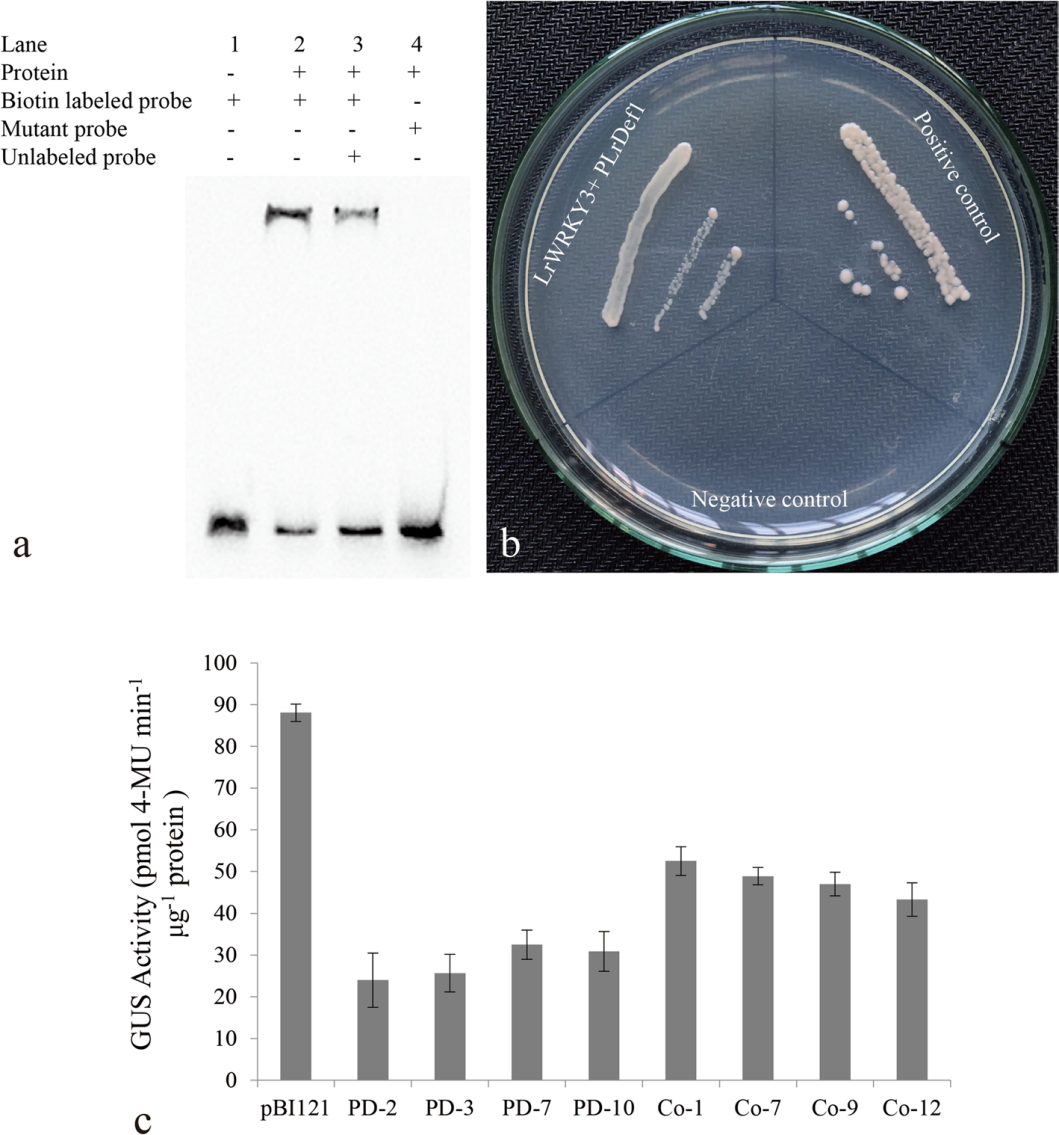
Analysis of the interaction between LrWRKY3 and *LrDef1* promoter. **a** EMSA revealed that the LrWRKY3 specifically bound to W box. 1, reaction solution containing only free probes; 2, LrWRKY3 can interact with biotin labelled probes containing the W box sequence; 3, LrWRKY3 can both interact with biotin labelled probes containing the W box sequence and unlabeled probes containing the W box sequence; 4, LrWRKY3 can’t interact with mutant probes; Complex: the combination of LrWRKY3 recombinant protein and probes; Free probe: the unbound probes. **b** Analysis of the transactivation effect of LrWRKY3 on *LrDef1* promoter. LrWRKY3 + *pLrDef1*: transcriptional activity analysis of pGADT7-*LrWRKY3* interaction with *pLrDef1*; Positive control: pGADT7-*p53* can interact with pAbAi-*p53* as a positive control; Negative control: pAbAi empty vector was used a negative control. **c** GUS activity was higher in transgenic tobacco lines co-expressing of *LrWRKY3* and *LrDef1* promoter than in the transgenic tobacco lines expressing of *LrDef1* promoter. The results were shown as average values calculated from three replicates and calculated by the 2^-ΔΔCt^ method and analyzed by the Student’s *t* test. pBI121: pBI121-*GUS* transgenic tobacco; PD-2/3/7/10: *pLrDef1* transgenic tobacco lines. Co-1/7/9/12: *LrWRKY3*/*pLrDef1* transgenic tobacco lines

The recombinant vector pET-32(a)-*LrWRKY3* was constructed and transformed into *E. coli* strain BL21 (DE3) for heterologous expression to obtain a LrWRKY3 recombinant protein. An electrophoretic mobility shift assay (EMSA) was used to analyze the binding between the LrWRKY3 recombinant protein and probes to reveal whether the specific binding site of LrWRKY3 is W box. As shown in Fig. 8a, lanes 1 and 2 on the EMSA gel both contained biotin-labeled probes designed from the *LeDef1* promoter fragment with a W box. Original image of Fig. 8a was shown in the supplementary information (Additional file 3). The band in lane 1, corresponding to biotin-labeled probe with no LrWRKY3 recombinant protein added, was not retarded on the gel. The presence of retarded bands in lanes 2 and 3 after the addition of LrWRKY3 indicates that LrWRKY3 was able to bind to the probes containing W box, which slowed their migration rates on the gel. The band in lane 3 was less delayed than the band in lane 2 because of competition due to the large number of unlabeled probes, which were 50 times more abundant than the biotin-labeled ones. Moreover, the LrWRKY3 protein was unable to bind to the mutant probe in lane 4 and thus no mobility shift was observed. These data fully illustrate that LrWRKY3 specifically binds to the W box.

### LrWRKY3 transcriptionally activated *LrDef1* in yeast cells

To determine whether LrWRKY3 has transcriptional activation activity, we integrated the ORF of *LrWRKY3* into the prey vector pGADT7 AD in a Y1H system followed by co-transformation with the recombinant bait vector pAbAi-*pLrDef1* in Y1HGold yeast cells, with the pAbAi-*p53* plasmid used as positive control and the pAbAi empty vector as negative control. Yeast cells co-transformed with pGADT7 AD-*LrWRKY3* and pAbAi-*pLrDef1* vectors were able to grow normally on auxotrophic SD/−Leu/AbA solid medium (Fig. 8b). In contrast, yeast cells co-transformed with pGADT7 AD-LrWRKY3 and bait empty vectors could not grow on the solid medium. These results indicate that pGADT7 AD-LrWRKY3 could be integrated into the yeast genome containing bait plasmid to produce a fusion protein, Gal4-LrWRKY3, capable of recognizing and activating the W box of *pLrDef1* in yeast cells. The Gal4-LrWRKY3 protein activated the expression of *UR1-C* in the recombinant bait plasmid pAbAi*-pLrDef1*, which enabled yeast cells to grow normally on SD/−Leu/AbA medium. This outcome suggests that the LrWRKY3 protein can specifically bind to the *LrDef1* promoter in yeast and has trans-activation activity.

### LrWRKY3 positively regulated the expression of *F. oxysporum-*resistance gene *LrDef1* in tobacco

Next, *LrWRKY3* and the *LrDef1* promoter were co-expressed in tobacco to explore the effect of LrWRKY3 on transcriptional activity of *LrDef1* promoter. According to a GUS activity analysis, the β-glucuronidase (*GUS*) gene had the highest activity in pBI121-*GUS* transgenic tobacco, approximately 88 pM 4-MU min^− 1^ μg^− 1^ (Fig. 8c). At the same time, GUS activity in *LrWRKY3*/*pLrDef1* transgenic tobacco was significantly higher than that in *pLrDef1* transgenic tobacco. The average GUS activity of four *pLrDef1* transgenic tobacco lines (PD-2/3/7/10) was approximately 24, 26, 33, and 31 pM 4-MU min^− 1^ μg^− 1^, respectively. The average GUS activity of four *LrWRKY3*/*pLrDef1* co-expressing transgenic tobacco lines (Co-1/7/9/12) was higher than 40 pM 4-MU min^− 1^ μg^− 1^, namely, approximately 52, 48, 46, and 42 pM 4-MU min^− 1^ μg^− 1^, respectively. Noteworthily, the average activity of Co-1 was approximately 1.7-fold higher than that of PD-7. These results indicate that the specific bindings of LrWRKY3 with W box in *LrDef1* promoter activated the expression of downstream reporter gene *GUS* driven by *LrDef1* promoter, thereby enhancing GUS activity.

## Discussion

A large amount of evidence indicates that WRKY TFs play important roles in regulating plant resistance to pathogenic fungal infection. To explore the function of WRKY TFs in *L. regale*, we isolated the WRKY family member gene *LrWRKY3* from *L. regale* in the present study. Structural analysis revealed that *LrWRKY3* contains a highly conserved ‘WRKYGQK’ short peptide and a C_2_H_2_ zinc finger, thus indicating that this gene belongs to the IIa WRKY TF group. Nuclear localization is essential for TFs to activate or inhibit expression of their target genes under stress conditions. In subcellular localization experiments, the green fluorescence signal of the LrWRKY3-GFP fusion protein was detected in the nuclei of onion epidermal cells. These results indicate that LrWRKY3 is a nuclear-localized protein that possibly regulates cellular transcriptional reprogramming under biotic stress.

Gene overexpression and gene silencing are commonly used to investigate the role of WRKY TFs in plant defense. Transgenic Arabidopsis plants overexpressing grapevine (*V. davidii*) WRKY53 show strong disease resistance to *Coniella diplodiella*, *Pseudomonas syringae*, and *G. cichoracearum* [17]*.* Overexpression of *SpWRKY3* in currant tomato (*Solanum pimpinellifolium*) positively modulates defense response to *Phytophthora infestans*, as indicated by a reduced number of necrotic cells, lesion size, and disease index; however, the resistance is weakened by *SpWRKY3* silencing [18]. The transient silencing of strawberry (*Fragaria × ananassa*) *WRKY1* reduces sensitivity to *Colletotrichum acutatum* and has indicated that *FaWRKY1* plays a negative regulatory role in strawberry resistance to *P. scutellum*. Even more interesting, *FaWRKY1* positively regulates resistance to *P. syringae* in *Arabidopsis* [19]. In the present study, reverse genetics was used to verify the function of *LrWRKY3*. Overexpression of *LrWRKY3* in tobacco enhanced resistance to *F. oxysporum*. At the same time, the transient expression of *LrWRKY3* RNAi vector in *L. regale* scales increased decay and disease area, which indicates that the decreased expression of *LrWRKY3* in *L. regale* scales increased susceptibility to *F. oxysporum*. These data clearly indicate that *LrWRKY3* is an important positive regulator in defense responses of *L. regale*.

Plant defense systems rely on a complex signal regulatory network. Phytohormones play a crucial role, with SA and JA, in particular, serving as important immune response regulators [20]. WRKY TFs are important regulatory nodes in SA, JA, and ETH signaling pathways [21]. *OsWRKY4* enhances rice resistance to the necrotrophic pathogen *Rhizoctonia solani* and participates in JA-mediated pathway [22]. Overexpression of *NtWRKY50* in tobacco alters SA and JA contents, increases expression of defense-related genes, and enhances resistance to *R. solanacearum* [23]. In our previous study, SA, MeJA, H_2_O_2_, ETH, and *F. oxysporum* treatment induced the expression of *LrWRKY3* [12]. In the SA-mediated signaling pathway, PR1, which is downstream of NPR1, is positively regulated by NPR1 to acquire resistance [24]. In the present study, transcription levels of *NtNPR1* and *NtPR1* were significantly increased in *LrWRKY3* transgenic tobacco lines. In addition, expression levels of JA biosynthetic pathway-related genes (*NtLOX*, *NtAOC*, *NtOPR*, *NtAOS*, *NtKAT*, *NtPACX*, and *NtJMT*) were significantly higher in *LrWRKY3* transgenic tobacco lines compared with WT tobacco. These results suggest that LrWRKY3 participates in JA- and SA-mediated signal transduction pathways, which are essential in the immune response of *L. regale* against Fusarium wilt.

The overexpression of *OsWRKY80* in rice significantly enhances resistance to sheath blight and up-regulates transcription levels of some disease resistance-related genes, including *PR1a*, *PR1b*, *PR5*, and *PR10* in transgenic lines compared with WT plants. The RNAi of *OsWRKY80* significantly reduces sheath blight resistance, however, and the above-mentioned disease resistance-related genes are depressed in *OsWRKY80* RNAi lines [25]. In the present study, we also analyzed the expression levels of three *PRs* (*NtGlu2*, *NtCHI*, and *Ntosmotin*) and three *SODs* (*NtSOD*, *NtCu-ZnSOD*, and *MnSOD*). These genes were significantly up-regulated in the *LrWRKY3* transgenic tobacco lines compared with WT, which suggests that *LrWRKY3* modulates the expression of defense-related genes during response to *F. oxysporum*.

Antimicrobial peptides, including defensins, are the main components of plant innate immune system. Plant defensins, a class of small, cysteine-rich alkaline cationic peptides, are the first line of defense against pathogen invasion [26]. These compounds can bind to fungus-specific membrane components, leading to invagination of the cell membrane of pathogenic fungi; they can also be intercalated or transported into the cell membrane to give rise to a series of biochemical changes [27]. Various plant defensins have been introduced into plants to enhance their resistance. For instance, overexpression of a *Panax notoginseng* defensin-like gene (*PnDEFL1*) in tobacco enhanced resistance to *F. solani*; moreover, the PnDEFL1 recombinant protein showed antifungal activity against *F. solani*, *F. oxysporum*, *Botrosphaeria dothidea*, and *S. sclerotiorum* in vitro [28]. As another example, overexpression of the *NmDef02* reduced the susceptibility of soybean (*Glycine max*) to *Phakopsora pachyrhizi* and *C. truncatum* and improved resistance against diseases [29]. In the present study, a *F. oxysporum*-responsive defensin gene, *LrDef1*, was isolated from *L. regale*. Bioinformatics analysis revealed that LrDef1 possesses a signal peptide corresponding to the characteristics of plant defensins, and subcellular localization experiments confirmed that LrDef1 encodes a cell wall protein. Further analyses revealed that LrDef1 is an antifungal protein, with the recombinant protein LrDef1 able to inhibit the mycelial growth of *F. oxysporum*, *F. solani*, and *Alternaria alternata.* In addition, the overexpression of *LrDef1* in tobacco enhanced the resistance of tobacco to *F. oxysporum*. We thus conclude that *LrDef1* is a *F. oxysporum*-resistance gene in *L. regale*.

The DNA binding domain can bind to the W box element [(C/T) TGAC (T/C)] in promoter of target genes of WRKYs, with WRKYs in turn activating or inhibiting the expression of downstream genes [30]. The currant tomato *WRKY1* activates the non-coding RNA lncrna33732 through sequential-specific interaction with the W box element in promoter; moreover, lncrna33732 can induce the expression of respiratory oxidase and increase the accumulation of H_2_O_2_, thereby enhancing currant tomato resistance to *P. infestans* [31]. In the present study, we obtained the *LrDef1* promoter sequence (850 bp) by TAIL-PCR, and bioinformatics analysis indicated that the *LrDef1* promoter sequence had a binding site specific to WRKYs, the W box. The results of subsequent EMSA and yeast one-hybrid experiments leave little doubt that LrWRKY3 can specifically bind to the W box of *LrDef1* promoter and has transcriptional activation activity in yeast cells. In addition, GUS activity was significantly enhanced in *pLrDef1*-*GUS/LrWRKY3* co-expressed tobacco compared with transgenic tobacco expressing *pLrDef1*-*GUS* alone. All these data strongly support the hypothesis that *LrWRKY3* has a transcriptional regulatory effect on the defensin gene *LrDef1*, an important resistance gene in response to *F. oxysporum* infection.

The present work will be subsequently explored for further research. Firstly, the *LrWRKY3* transgenic tobacco lines will be subjected to transcriptome sequencing and determination of hormone content in order to further reveal the molecular net regulated by *LrWRKY3* and verify the crosstalk between LrWRKY3 and hormone-mediated signaling pathways. In addition, it is urgent to establish a rapid genetic transformation method of *L. regale*, and we will be able to thoroughly understanding the regulatory mechanism of WRKY TFs in *L. regale* during response to Fusarium wilt in the near future.

## Conclusion

A nuclear-localized IIa WRKY TF gene, *WRKY3*, was isolated from *L. regale*. Overexpression of *LrWRKY3* in tobacco improved resistance to *F. oxysporum*; however, RNAi of *LrWRKY3* in *L. regale* scales conferred sensitivity to *F. oxysporum* infection*.* Moreover, *LrWRKY3* activated JA and SA signal pathways and up-regulated the expression levels of *PRs* and *SODs* in the transgenic tobacco lines. In addition, *LrWRKY3* showed transactivation on a *F. oxysporum*-resistance gene, *LrDef1*. We thus conclude that LrWRKY3 modulates the antimicrobial peptide gene *LrDef1* and JA/SA signal pathways in *L. regale* during response to Fusarium wilt.

## Experimental procedures

### Fungal and plant materials

Wild *L. regale* was collected in Wenchuan County, Sichuan Province, China, and was identified by our research group through referring to a *L. regale* specimen (Kun 222,503) preserved in the herbarium of Kunming Institute of Botany, Chinese Academy of Sciences, and then was further confirmed by Prof. Zhineng Li from Southwest University. The *L. regale* was planted in a greenhouse (25 °C, 60% relative humidity) at Kunming University of Science and Technology. Wild type tobacco (*N. tabacum*) seeds were provided by Dr. Guanze Liu from Yunnan Agricultural University, Kunming, China. Sterile tobacco seedlings were cultured in a climatic cabinet (25 °C, 16 h light/8 h dark cycle) and used for genetic transformations. A *F. oxysporum* strain from a diseased lily with typical symptoms of root rot was characterized and preserved by our research group. *F. oxysporum*, *F. solani*, and *A. alternata* were conserved at 4 °C and activated through culturing on potato dextrose agar medium plate for one week at 28 °C before use.

### Cloning of *LrWRKY3* and *LrDef1*

Total RNA was extracted from *L. regale* roots, and cDNA was synthesized with a reverse transcription kit (Promega, USA) to isolate *LrWRKY3* and *LrDef1*. The methods used for RNA extraction and cDNA synthesis were the same as those detailed in Liu et al. [32]. The ORFs of *LrWRKY3* and *LrDef1* were cloned by PCR with specific primers (Table S1), and the PCR products were cloned into pGEM-T vector (Promega). The gene-specific primer sequences were given in the supplementary information (Additional file 2, Table S2). Positive *E. coli* clones containing pGEM-T-*LrWRKY3* and pGEM-T-*LrDef1* were confirmed by PCR.

### Quantitative reverse transcription PCR (qRT-PCR)

The expression patterns of *LrDef1* in *L. regale* were analyzed by qRT-PCR. The roots of healthy *L. regale* plants were injured and then infected with *F. oxysporum* (2 × 10^6^ spores/mL) for 30 min. The roots were collected 24, 48, and 72 h after inoculation. The mimic inoculation with sterile distilled water was used as a control. Roots, leaves, stems, flowers, and scales of *L. regale* under normal growth conditions were collected to analyze the expression pattern of *LrDef1*. Total RNA was extracted from each sample and used for cDNA synthesis. qRT-PCR amplification conditions and instrumentation were the same as in Liu et al. [32]. The expression level of *L. regale* glyceraldehyde-3-phosphate dehydrogenase (*LrGAPDH*, GenBank no. JZ391059) was used as an internal control to standardize different RNA samples. The qRT-PCR gene-specific primers sequences were given in the supplementary information (Additional file 2, Table S3). Three biological replicates were performed for each qRT-PCR assay, and the 2^−ΔΔCt^ method was used to calculate relative gene expression levels.

### Subcellular localization analysis

The subcellular locations of LrWRKY3 and LrDef1 were predicted using the PSORT online program (https://www.genscript.com/psort.html) and verified by transient expression of a GFP-tagged fusion protein in onion epidermal cells. The ORFs of *LrWRKY3* and *LrDef1* without stop codons were digested by restriction enzymes and then ligated to a pBIN M-gfp5-ER vector using T4 ligase for fusion with *GFP*. The pBIN m-gfp5-ER-*LrWRKY3* and pBIN m-gfp5-ER-*LrDef1* recombinant plasmids were individually transferred into *Agrobacterium tumefaciens* EHA105 via the freeze-thaw method. An empty pBIN m-gfp5-ER vector served as a control. The transformation protocols into onion epidermis are detailed in Qiu et al. [33]. Confirmation of LrWRKY3 and LrDef1 subcellular localizations was performed with a confocal laser scanning microscope (Nikon, Japan). The GFP fluorescence was detected using FITC channel with excitation wavelength of 488 nm, and the red fluorescence of PI was detected using Cy5 channel with excitation wavelength of 655 nm. The localizations of LrWRKY3 and LrDef1 were determined according to the GFP fluorescence distribution of two fusion proteins in the onion cells.

### Construction of *LrWRKY3* and *LrDef1* plant overexpression vectors and genetic transformation of tobacco

The pCAMBIA2300s vector was used to construct plant overexpression vectors for *LrWRKY3* and *LrDef1*. The ORFs of *LrWRKY3* and *LrDef1* were respectively obtained from pGEM-T-*LrWRKY3* and pGEM-T-*LrDef1* by double enzyme digestion. The enzyme digestion products were then ligated with the pCAMBIA2300s vector, which was digested with the same restriction enzyme. The ligation products were transferred into *E. coli* DH5α competent cells, and positive clones were screened by PCR. The pCAMBIA2300s-*LrWRKY3* and pCAMBIA2300s-*LrDef1* plasmids were separately transferred into *A. tumefaciens* LBA4404. Positive clones were identified by PCR and used for genetic transformation of tobacco according to Horsch et al. [34]. The positive transgenic tobacco seedlings were confirmed by PCR with two pairs of gene-specific primers, with the WT included in the PCR analysis as a negative control. In addition, some transgenic tobacco plants were randomly selected for self-breeding cultivation T_2_ generation in a greenhouse.

### Gene expression and disease resistance analyses of transgenic tobacco

Transcription levels of *LrWRKY3* and *LrDef1* in young leaves of T_2_ generation transgenic tobacco were analyzed by qRT-PCR, with the tobacco actin gene (*NtACT*, GenBank no. AB158612.1) used as an internal reference. Four lines of T_2_ generation transgenic tobacco were selected to study their resistance to *F. oxysporum* infection. The leaves of WT tobacco and T_2_ generation transgenic tobacco seedlings were injured with sandpaper and inoculated with 100 μL *F. oxysporum* spore suspension (2 × 10^6^ spores/mL). The infected leaves were laid flat on wet filter paper to maintain humidity and placed in an illumination incubator. The injured roots of T_2_ generation *LrWRKY3* transgenic tobacco and WT tobacco seedlings were immersed in *F. oxysporum* spore suspension (2 × 10^6^ spores/mL) for 30 min and then transferred to half-strength Murashige–Skoog liquid culture medium. The inoculated tobacco samples were collected after 7 days, and disease symptoms were recorded with a digital camera (Nikon). Lesion areas were measured with Photoshop. The leaves of *LrDef1* T_2_ generation transgenic tobacco were inoculated with *F. oxysporum* for disease resistance analysis using the above-mentioned approaches.

### Measurement of expression levels of JA biosynthesis, SA signaling pathway, and defense-related genes in *LrWRKY3* transgenic tobacco

qRT-PCR was used to analyze transcription levels of JA biosynthetic pathway-related genes (*NtLOX*, *NtAOC*, *NtOPR*, *NtAOS*, *NtKAT*, *NtPACX*, and *NtJMT*), SA signaling pathway-related genes (*NtPR1* and *NtNPR1*), *PRs* (*NtGlu2*, *NtCHI*, and *Ntosmotin*), and *SODs* (*NtSOD*, *NtCu-ZnSOD* and *MnSOD*) in four *LrWRKY3* transgenic tobacco lines. The tobacco *actin* was used as a reference gene. Gene-specific primers were designed according to sequences downloaded from NCBI (https://www.ncbi.nlm.nih.gov/) and are listed in Table S2.

### Transient expression of *LrWRKY3* RNAi construct in *L. regale* scales

Gene-specific primers (Table S1) of *LrWRKY3* with an attB linker were designed to amplify a RNAi fragment (410 bp). The *LrWRKY3* RNAi PCR products were recombined with pHellsgate2 vectors using Gateway BP Clonase II enzyme mixture (Invitrogen, USA). The BP reaction mixture was then transformed into *E. coli* DH10B competent cells. The pHellsgate2-*LrWRKY3* recombinant plasmids were further transformed into *A. tumefaciens* EHA105, and the positive clones were identified by PCR. The scales of healthy *L. regale* plants were wounded with sandpaper, and *A. tumefaciens* suspension (50 μL) containing pHellsgate2-*LrWRKY3* or pHellsgate2 empty vector was then added. The infected scales were placed in a climate chamber at 28 °C for 24 h for the transient expression of RNAi vectors in *L. regale* scales, with subsequent *F. oxysporum* inoculation with spore suspension (2 × 10^6^ spores/mL) at the wound sites. Symptoms and lesion areas of lily scales were recorded and measured after inoculation for 72 h. In addition, the expression levels of *LrWRKY3* in scales were measured by qRT-PCR.

### Prokaryotic expression and antifungal activity analysis of LrDef1 recombinant protein

The SignalP-5.0 program (https://www.cbs.dtu.dk/services/SignalP/) was used to predict the signal peptide in LrDef1. The PCR-amplified *LrDef1* ORF without the signal peptide-encoding sequence but containing the restriction sites of *Eco*RV and *Eco*RI was subcloned into the prokaryotic expression vector pET-32(a) to produce 6 × His-labeled fusion protein. The pET-32(a)-*LrDef1* construct was then transformed into *E. coli* BL21 (DE3) for heterogenic expression. The LrDef1 recombinant protein was purified by Ni-NTA-sepharose affinity chromatography. *F. oxysporum*, *F. solani*, and *A. alternata* were used to analyze the antifungal effects of the LrDef1 recombinant protein using the method described by Taif et al. [35].

### Isolation of the LrDef1 promoter fragment

The *LrDef1* promoter fragment was isolated using a Genome Walking kit (Takara, Japan) according to the kit instructions. Two nested gene-specific primers and a degenerate primer were used in two successive PCR rounds to amplify the *LrDef1* promoter region. The secondary PCR products were cloned into pGEM-T vector and then transformed into *E. coli* DH5α. Positive clones were selected for sequencing. In addition, the *cis*-elements in promoter were predicted using the PlantCARE program (http://bioinformatics.psb.ugent.be/webtools/plantcare/html/).

### Prokaryotic expression of the LrWRKY3 recombinant protein and EMSA

A pair of gene-specific primers (Table S1) were designed to amplify the *LrWRKY3* ORF, which was then subcloned into the pET-32(a) vector. The recombinant pET-32(a)-*LrWRKY3* plasmids were further transformed into *E. coli* BL21 (DE3) to express the recombinant protein containing a 6 × His tag. Detailed protocols on the recombinant protein denaturation and renaturation of inclusion body protein are given in Zhao et al. [36].

The EMSA was used to confirm the specific binding of LrWRKY3 recombinant protein with the *LrDef1* promoter fragment. A probe containing the W box sequence of *LrDef1* promoter was designed and labeled with biotin (Sangon Biotech) and is hence referred to as the biotin-labeled probe (Table S1). In addition, a W box sequence containing a mutation was labeled with biotin and used as a mutant probe. Moreover, a probe without biotin labeling, designated the unlabeled probe, was used as the competitor. The biotin-labeled and mutant probes were diluted to a concentration of 0.01 μM, and the final concentration of unlabeled probe was 0.5 μM. Equivalent amounts of sense and antisense fragments of these probes were annealed to synthesize double-stranded DNA. After addition of the biotin-labeled probes, the EMSA reaction mixture including 0.5 μg purified LrWRKY3 protein was incubated for 20 min at 25 °C and then subjected to gel electrophoresis. For the competition experiment, the unlabeled probe was added as a competitor in the binding reaction and incubated for 10 min before addition of the biotin-labeled probe. The reaction mixture was incubated at room temperature for another 15 min prior to loading onto the gel. EMSA was performed according to the standard procedures of a Light Shift Chemiluminescent EMSA kit (Pierce, USA).

### Yeast one-hybrid assay

A yeast one-hybrid system (Takara) was used to analyze the transactivation of *LrDef1* by LrWRKY3. The 500-bp *LrDef1* promoter fragment containing a W box was ligated to the bait vector pAbAi, and the ligation product pAbAi-*pLrDef1* was then transformed into *E. coli* DH5α. Positive clones containing the pAbAi-*pLrDef1* recombinant plasmids were selected by PCR, and the recombinant plasmids were transformed into yeast cell Y1Hgold to obtain the yeast bait strains according to the method described in Li et al. [12]. In addition, the *LrWRKY3* ORF was ligated to the prey vector pGADT7 AD and transferred into *E. coli* DH5α. The yeast prey recombinant vector pGADT7 AD-*LrWRKY3* was then transferred into bait strain pAbAi-*pLrDef1-*Y1HGold competent cells. Moreover, the yeast prey empty vector pGADT7 AD was transferred into bait strain pAbAi- *pLrDef1-*Y1HGold competent cells as a negative control, and the pGADT7 AD-*P53* vector (kit provided, Takara) was transferred into bait vector pAbAi-*p53*-Y1HGOLD competent cells as a positive control. The appropriate inhibitory concentration of aureobasidin A (AbA) against the bait reporter strains was determined according to the manufacturer’s instructions (Takara). The resulting three groups of yeast cells were streaked onto SD/−Leu/AbA solid medium, and the plates were placed in a 30 °C incubator for 2–3 days to observe whether LrWRKY3 activated the expression of *LrDef1* promoter.

### Co-expression of the *LrDef1* promoter and *LrWRKY3* in tobacco

To verify whether the LrWRKY3 TF has a regulatory effect on *LrDef1* in plants, the *LrDef1* promoter was transformed into *LrWRKY3* transgenic tobacco to obtain the co-expression tobacco. The CaMV 35 s promoter in pBI121 vector was replaced by *LrDef1* promoter to construct a plant expression vector that included a *GUS* gene driven by *LrDef1* promoter. The pBI121-*pLrDef1*-*GUS* plasmids were transformed into *A. tumefaciens* LBA4404 competent cells and then transformed into WT or *LrWRKY3* T_2_ generation transgenic tobacco leaf discs. In addition, the WT tobacco leaf disks were transformed with the empty pBI121-*GUS* vector as a control. Positive transgenic tobacco plants were identified by PCR with *GUS*-specific primers. A fluorescence spectrophotometer (Hitachi f-4600, Japan) was used to determine the GUS enzyme activity (pM 4-MU min^− 1^ μg^− 1^ protein) of positive transgenic tobacco as described by Chen et al. [37].

### Data analyses

Gene expression levels, lesion sizes, fungal growth inhibition zones, and GUS activities were calculated as means plus standard deviations. Student’s *t-*test was performed using SPSS software (version 17.0) to reveal statistical differences between treatments, inoculations, and controls.

## Supplementary Information

**Additional file 1. **The gene sequences of *LrDef1* and *pLrDef1*.

**Additional file 2: Table S1.** Prediction results of *cis*-acting elements in the promoter sequence of *LrDef1*. **Table S2.** The primers sequences used for function analysis of *LrWRKY3* and *LrDef1*. **Table S3.** The qRT-PCR gene-specific primers sequence.

**Additional file 3: Fig. S1.** The original gel image of Fig. 6a. **Fig. S2.** The original gel image of Fig. 6b. **Fig. S3.** The original gel image of Fig. 8a.

## Data Availability

The original contributions presented in the study are publicly available. The data can be found in the NCBI (https://www.ncbi.nlm.nih.gov/): *LrWRKY3* accession number: MW125548, *LrDef1* accession number: MZ872924, *pLrDef1* accession number: MZ872925.

## Abbreviations

TFs: Transcription factors
SA: Salicylic acid
MeJA: Methyl jasmonate
ETH: Ethylene
H_2_O_2_: Hydrogen peroxide
JA: Jasmonic acid
RNAi: RNA interference
ORF: Open reading frame
GFP: Green fluorescent protein
WT: Wild type
PR: Pathogenesis-related protein
SOD: Antioxidant stress-related superoxide dismutase
GUS: β-glucuronidase
Co: Co-expression
EMSA: Electrophoretic mobility shift assay
AbA: Aureobasidin A
