# Supplementary material for: Lilium regale Wilson WRKY3 modulates an antimicrobial peptide gene, LrDef1, during response to Fusarium oxysporum
Source: BMC Plant Biol. 2022 May 24;22:257. doi: 10.1186/s12870-022-03649-y (PMC9128230; doi:10.1186/s12870-022-03649-y)
Supplement: Supplementary file 1 — Additional file 1. The gene sequences of LrDef1 and pLrDef1. [file 12870_2022_3649_MOESM1_ESM.docx]

>LrDef1 (NCBI accession number: MZ872924)

TCGTCGTCCCCATCTCAGTGGCCGACTATCTCTTGCAATGGCGAAGCTTCCCACCATCCTGCTGCTCTTGTTCCTTGTCATGGCCACTGAGATGGGGACGACGACGGTGGAGGCGAGGACATGCCTGTCGCAGAGCCACAAGTTCAAGGGGACCTGTTTGAGGGCGGCCAACTGTGCTAGTGTCTGCCAGACGGAGGGATTCAAAGGAGGGGTTTGCGAGGGCATCCGCCGCCGTTGCTTCTGCGAAGCCGACTGTCACTGATGCCTGAGTTCTTGGCTTTAATAAGTAATGTCGGACTATCCGAGAAGAATAAGATGGACCTGGTGTTGTTGGTTTTACAGTCTCTTCTTCGGTGTGGGGACTCGGTACTTTCATCTAGGTTTCTGATATGTAGTTGTTCATGTCTGGGTTGAGCTGTAGGGCTGTGTGCTGTAGTTGGATTTGTAGTGGAGTAAGTTCTCTTGTATTTGATTTGTAGTGGAGTAAGTTCTCTTGTATTT

>pLrDef1 (NCBI accession number: MZ872925)

ATGTTTGGTCTAAACAATCATGATTACACACCCCTACTAAATTATATAACGCATTGCTCCCACAATGATCAGTGTCCCCATGCACATCAATATTATCTACCAACAAACAACTCTTACATTTTTTTTGTGTGTTTTTATCTCATCCGTCATTGACCACCCATAATAAGCTTTGATCCACCCACTCTATTTTTTCTTTGTTTTTTGCTTTCCTCTATCTTATTCTATCACTTTTTATTTTAAGTTGTGCACACCAAACATAATGCTAGAGTATTTTTTTTTTAATGTTTTCCTCCTTCAGGAGATCATATATGTGACATATTCAATAACTACTTAAAAGATGTGTGTTCTAACAAATGAGAACCATATTAATTATAAAAATTCTGATGTGAGGGATTATAGGACTTCGTGAGGATCCATAATGAAACTCAAAGTTGCATAGCGATGAGCCATCTAAGATCTGTTAGGAATTTATAGATGAAAAATATGTAATTTTGAATGAACTAACATTTAATTGCTCACGATGTGGTGGAAAGATACTGTCCACAATTTTCACTGGTCCGAGACTTGATAAAATGTAAAAAATTACAGACATGACAAAATGTATGAGTGAGCACGTCAGGCTTGATTCCCTCTCTCTTTGAAATCAATGATAAATAGACGACACACAGACAAAACAATCCCATCAGACATGTCGCTGCCACTGCCATCGCCTACTTTGTCTGACCGTATCCTCGTGACCCACGGGTTATCGAGACCCATCCCTCATCTAGGGTTTCAGTTTTCAAACTCTATCCTTCAACCAAAACCCTGGCTCCATCTATAAAAACCACTCCCCCACCACTCCCACCTA
