# Supplementary material for: Lilium regale Wilson WRKY3 modulates an antimicrobial peptide gene, LrDef1, during response to Fusarium oxysporum
Source: BMC Plant Biol. 2022 May 24;22:257. doi: 10.1186/s12870-022-03649-y (PMC9128230; doi:10.1186/s12870-022-03649-y)
Supplement: Supplementary file 2 — Additional file 2: Table S1. Prediction results of cis-acting elements in the promoter sequence of LrDef1. Table S2. The primers sequences used for function analysis of LrWRKY3 and LrDef1. Table S3. The qRT-PCR gene-specific primers sequence. [file 12870_2022_3649_MOESM2_ESM.docx]

Table S1. Prediction results of *cis*-acting elements in the promoter sequence of *LrDef1*

| The name of *cis*-element | Sequence | Elements function | Position(bp) |
| --- | --- | --- | --- |
| ROOTMOTIFTAPOX1 | ATATT | Root-specific regulatory elements | -89~-93  -315~-319  -362~-366 |
| CAREOSREP1 | CAACTC | Regulation of GA induced gene expression | -107~-111 |
| OSE2ROOTNODULE | CTCTT | Tissue specific element | -110~-114  -633~-647 |
| WBOXATNPR1 | TTGAC | SA response element | -149~-153 |
| W-box | C/TTGACC/T | ET, SA, mesa response elements, WRKY transcription factor binding elements, and defense gene response elements induced by fungi | -149~-154 |
| WBOXNTERF3 | TGACC | Injury stress response element | -150~-154  -720~-724  -734~-738 |
| -10PEHVPSBD | TATTCT | Response optical control element | -217~-222 |
| WRKY71OS | TGAC | GA3 response element | -311~-314  -591~-594  -720~-723  -734~-737 |
| EBOXBNNAPA | CAAATG | ABA response element | -350~-355 |
| MYCCONSENSUSAT | CAAATG | Participate in cold stress response | -350~-355 |
| MYBCORE | CTGTTA | Water stress response element | -457~-462 |
| BIHD1OS | TGTCA | Disease resistance response related elements | -591~-595 |
| ABRELATERD1 | ACGTG | ABA response element,High salt and inductive response elements | -611~-615 |
| ACGTATERD1 | ACGT | Participate in dehydration response | -612~-615 |
| IBOXCORE | GATAA | Light adjusting element | -648~-652 |
| CAT-box | GCCAC | Meristem expression related elements | -696~-700 |
| MYB1AT | AAACCA | Participate in dehydration response | -823~-828 |
| ARE | AAACCA | Anaerobic induction element | -823~-828 |

Table S2. The primers sequences used for function analysis of *LrWRKY3* and *LrDef1*

| Use | Target genes | Primer sequences |
| --- | --- | --- |
| Amplified ORF | *LrWRKY3* | Forward:5' CTCTCATTCATGGAGAGCT 3'  Reverse:5' ACATACTCTGCCCATAATACT 3' |
|  | *LrDef1* | Forward:5' TCGTCGTCCCCATCTCAGTGG 3'  Reverse:5 'TCAAATACAAGAGAACTTAC 3' |
| Subcellular localization | *LrWRKY3* | Forward:5' CATATGGAGAGCTTCCCCCTACTCCTGA 3'  Reverse:5' CCCGGGAGCTCGAATAGACTTGCATTTGG 3' |
|  | *LrDef1* | Forward:5' TCTAGAATGGCGAAGCTTCCCACCAT 3'  Reverse:5' GGATCCGTGAAGTCGGCTTCGCAGAA 3' |
| Prokaryotic expression | *LrWRKY3* | Forward:5' GATATCATGGAGAGCTTCCCCCTACT 3'  Reverse:5' GGAATTCCTAGATCAAAAGCTCGAATAGACTT 3' |
|  | *LrDef1* | Forward:5' GATATCCTCTTGTTCCTTGTCATGGCCAC 3'  Reverse:5' GAATTCGTGACAGTCGGCTTCGCAGAAG 3' |
| Ectopic expression | *LrWRKY3* | Forward:5' GCTCTAGAGCATTCATGGAGAGCTTCCCCCTAC 3'  Reverse:5' GGAATTCCTGGTGCATTGGCTTATTATACTCAT 3' |
|  | *LrDef1* | Forward:5' GAATTCATGGCGAAGCTTCCCACCA 3'  Reverse:5' GGATCCACTCAGGCATCAGTGACAGTCG 3' |
|  | GUS | Forward: 5' CGGATACCCGTCCGCAAGT 3'  Reverse: 5' GTGTGAGCGTCGCAGAACATT 3' |
| RNAi | *LrWRKY3* | Forward:5' GGGGACAAGTTTGTACAAAAAAGCAGGCTGGGTTGAAGGAGGAGACCGTGGCT 3'  Reverse:5' GGGGACCACTTTGTACAAGAAAGCTGGGTAAGCTCGAATAGACTTGCATTTGGT 3' |
| Genome-walking | *pLrDef1* | Pa1:5' TCCTTTGAATCCCTCCGTCTGGCA 3'  Pa2:5' ATGACAAGGAACAAGAGCAGCAGGATG 3' |
| Amplified promoter | *pLrDef1* | Forward:5' GAGTACTCTAACGCATTGCTCCCACA 3'  Reverse:5' GCTCTAGAGCGGGTTTTGGTTGAAGGATAG 3' |
| EMSA | *LrWRKY3* | Forward:5' GCTCTAGAGCCCCTCTCATCTCTCCATCTTCCT 3'  Reverse:5' GGAATTCCATCCACTGCTCCATTTATAGCCTAC 3' |
|  | Probe | TTTATCTCATCCGTCATTGACCACCCATAATAAGCTTTGATCCACCCACT |
|  | Mutant probe | TTTATCTCATCCGTCATTAACCACCCATAATAAGCTTTGATCCACCCACT |
| Yeast one-hybrid | *LrWRKY3* | Forward:5' CCCAAGCTTGGGCATAATGGCAGTCGGTTCCT 3'  Reverse:5' TCCCCCGGGGGAGGTAGAAACGTCACAAGCAAAA 3' |
|  | *pLrDef1* | Forward:5' CAAGCTTCAGTGTCCCCATGCACATCAATAT 3'  Reverse:5' CCCGGGTCAAGTCTCGGACCAGTGAAAATTG 3' |

Table S3. The qRT-PCR gene-specific primers sequence

| Target genes | Primer sequences |
| --- | --- |
| *LrWRKY3* | Forward:5' GATCCTCCGAGACCAATGCCA 3'  Reverse:5' CAGCGATAGCCGTCGTCAAGA 3' |
| *LrDef1* | Forward:5' CACCATCCTGCTGCTCTTG 3'  Reverse:5' CCTTTGAATCCCTCCGTCT 3' |
| *NtAOC*  (AJ308487.1) | Forward:5' AAGAAGAGAATTGGAATAACGGCT 3'  Reverse:5' GATCCCTGAACGGCGATGT 3' |
| *NtAOS*  (AB778304.1) | Forward:5' CCACCAGTTGCTTCTCAATACGG 3'  Reverse:5' GAACTCATCGGGTCGGTCAAA 3' |
| *NtPACX*  (KJ730264.1) | Forward:5'AAGCAGCTAAGTTAAGGCATTTTGTA 3'  Reverse:5'GTTCAGTTTGAGCGTAGCACCCA 3' |
| *NtKAT*  (XM_016651715.1) | Forward:5'TTGTTGATCCAAAAACCGGAGA 3'  Reverse:5' TGGCTAGAGTTCCCAGCAGTAGTAG 3' |
| *NtJMT*  (XM_016639122.1) | Forward:5' TTGGGTACTGAAGCAAGGACAGC 3'  Reverse:5' GCTCCTCCCCATTAACGACAAC 3' |
| *NtOPR*  (XM_016592560.1) | Forward:5' CGTTCATTTGTAGTGGCGGAT 3'  Reverse:5' CCTCATAACCAAATCAGGATTAGAAA 3' |
| *NtLOX*  (X84040.1) | Forward:5' TGGTTATCTCCCTAATCGCCCTAC 3'  Reverse:5' CGAGGTAAAGTGTATCCGAAGAATG 3' |
| *NtKAT*  (XM_016651715.1) | Forward:5' TTGTTGATCCAAAAACCGGAGA 3'  Reverse:5' TGGCTAGAGTTCCCAGCAGTAGTAG 3' |
| *Ntosmotin*  (LOC107794478) | Forward:5' CGACTATCGAGGTCCGAAACAAC 3'  Reverse:5' ACGTACCCCTACCAGCAGCATT 3' |
| *NtGlu2*  (A16120.1) | Forward:5' TTGATGCCCTTTTGGATTCTATG 3'  Reverse:5' TTTCCAGGTTTCTTTGGAGTTCC 3' |
| *NtPR-1*  (X05454.1) | Forward:5' AGAACCTTTGACCTGGGACGAC 3'  Reverse:5' ATCCAACACGAACCGAGTTACG 3' |
| *NtCHI*  (A16119.1) | Forward:5' ACGGACCTTGTGGAAGAGCCAT 3'  Reverse:5' ATCCAACACGAACCGAGTTACG 3' |
| *NtSOD*  (EU342358.1) | Forward:5' TGAAGCTGGTGGTCAATACATGG 3'  Reverse:5' GAGACATTGTCTTATATTGGAAGAGGA 3' |
| *NtCu-ZnSOD*  (EU123521.1) | Forward:5' CATGGTGCTCCTGAAGATGAGGT 3'  Reverse:5' CAGCATTTCCAGTAGCTTTACTGAG 3' |
| *MnSOD*  (CAA32643.1) | Forward:5' GTGTGGCTTGGTGTGGACAAAG 3'  Reverse:5' CCTCAAAACAACATCAAATATCCCTG 3' |
| *NtACT*  (AB158612.1) | Forward:5' TCCCATTGAGCATGGAATAGTAAGC 3'  Reverse:5' TACATGGCAGGTACATTGAAAGTCT 3' |
| *LrGAPDH*  (JZ391059) | Forward:5' ACTTGGTTTCCACTGATTTCCTCG 3'  Reverse:5' CTTGCTAATGTGGCGGATGAGAT 3' |
