# Supplementary material for: Lilium regale Wilson WRKY3 modulates an antimicrobial peptide gene, LrDef1, during response to Fusarium oxysporum
Source: BMC Plant Biol. 2022 May 24;22:257. doi: 10.1186/s12870-022-03649-y (PMC9128230; doi:10.1186/s12870-022-03649-y)
Supplement: Supplementary file 3 — Additional file 3: Fig. S1. The original gel image of Fig. 6a. Fig. S2. The original gel image of Fig. 6b. Fig. S3. The original gel image of Fig. 8a. [file 12870_2022_3649_MOESM3_ESM.docx]

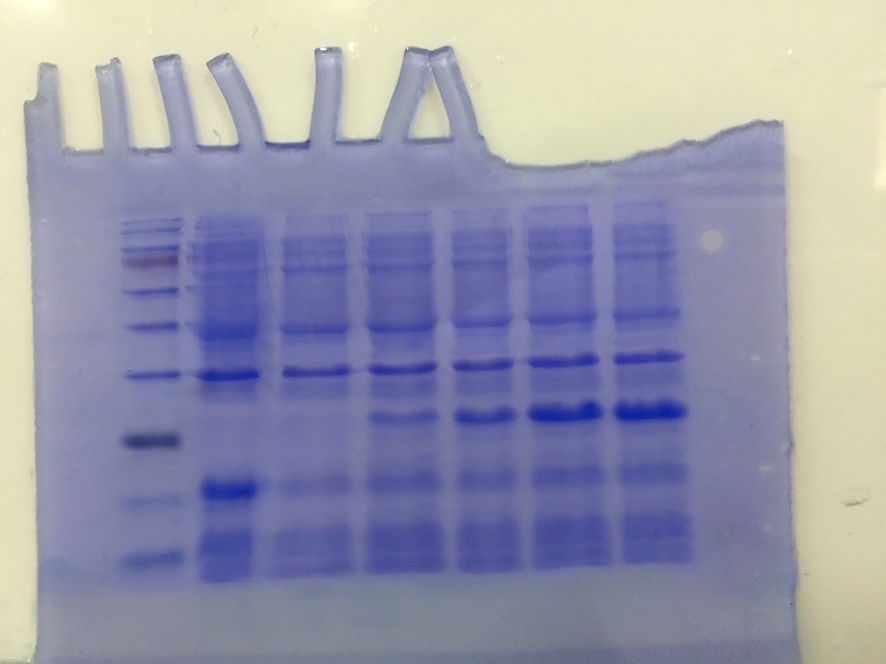


**Figure S1**. The original gel image of Figure 6a.


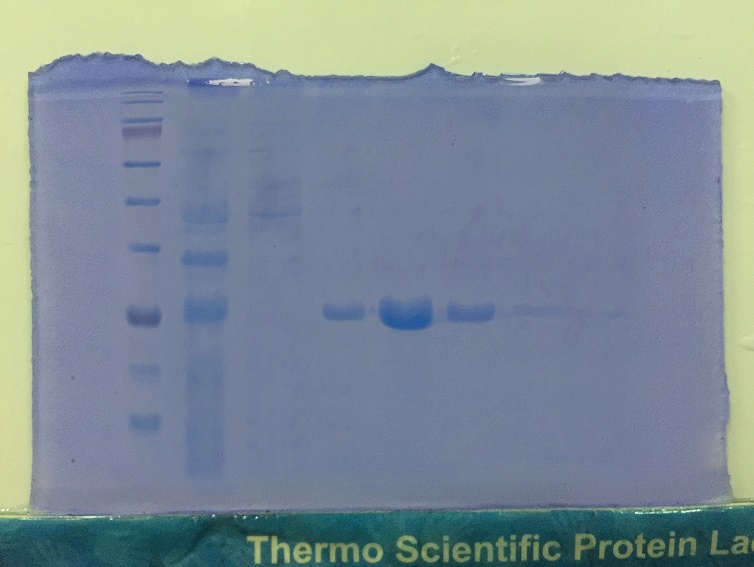


**Figure S2**. The original gel image of Figure 6b.


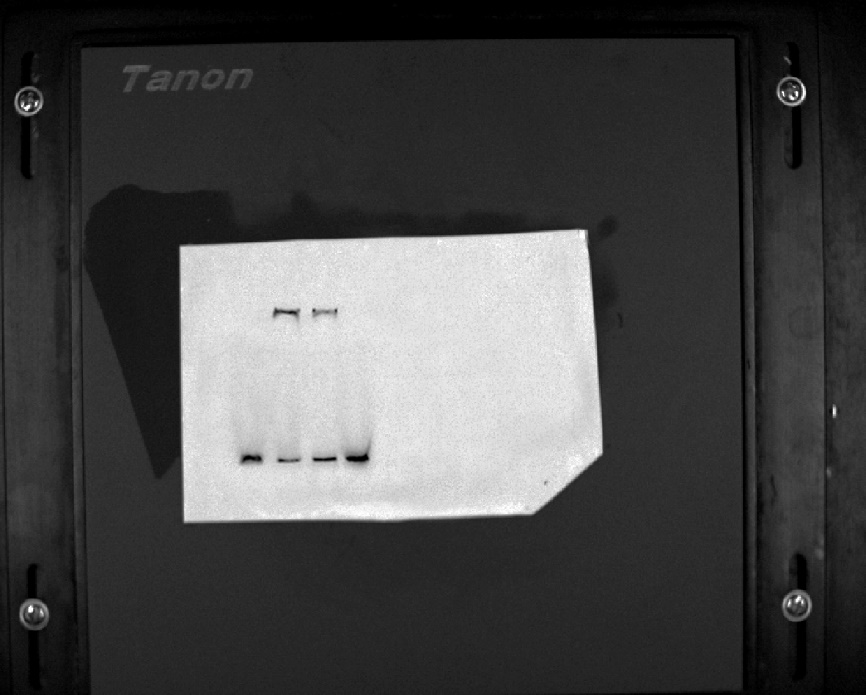


**Figure S3**. The original gel image of Figure 8a.
